# Risk of hepatocellular carcinoma in antiviral treatment-naïve chronic hepatitis B patients treated with entecavir or tenofovir disoproxil fumarate: a network meta-analysis

**DOI:** 10.1186/s12885-022-09413-7

**Published:** 2022-03-17

**Authors:** Ze-Hong Huang, Gui-Yang Lu, Ling-Xian Qiu, Guo-Hua Zhong, Yue Huang, Xing-Mei Yao, Xiao-Hui Liu, Shou-Jie Huang, Ting Wu, Quan Yuan, Ying-Bin Wang, Ying-Ying Su, Jun Zhang, Ning-Shao Xia

**Affiliations:** 1 State Key Laboratory of Molecular Vaccinology and Molecular Diagnostics, National Institute of Diagnostics and Vaccine Development in Infectious Diseases, Strait Collaborative Innovation Center of Biomedicine and Pharmaceutics, School of Public Health, Xiamen University, Fujian 361102 Xiamen, China; 2 The First Affiliated Hospital of Xiamen University, Xiamen, 361003 Fujian China; 3 The Research Unit of Frontier Technology of Structural Vaccinology of Chinese Academy of Medical Sciences, Xiamen, 361102 Fujian China

**Keywords:** Chronic hepatitis B, Hepatocellular carcinoma, Entecavir, Tenofovir, Cirrhosis

## Abstract

**Background:**

Long-term antiviral treatments are associated with a significantly lower hepatocellular carcinoma (HCC) incidence in chronic hepatitis B (CHB) patients by reducing HBV DNA concentrations. However, it is still controversial whether antiviral strategies affect HCC development in antiviral treatment-naïve CHB patients. This study aimed to estimate the incidence of HCC in antiviral treatment-naïve CHB patients who were treated with Entecavir (ETV) and Tenofovir Disoproxil Fumarate (TDF) and compare the efficacy of two treatment regimens in HCC reduction.

**Methods:**

The PubMed, Embase, China National Knowledge Infrastructure, and Wanfang databases were systematically searched until June 24, 2021. The pooled incidence and 95% confidence interval of HCC were calculated by the Freeman-Tukey double arcsine transformation method. The efficacies of ETV and TDF treatments in HCC reduction were compared through a network meta-analysis.

**Results:**

A total of 27 studies were identified as eligible for this systematic review. The incidence densities in the ETV and TDF treatment groups were 2.78 (95% CI: 2.21-3.40) and 2.59 (95% CI: 1.51-3.96) per 100 persons-year among patients with preexisting cirrhosis and 0.49 (95% CI: 0.32-0.68) and 0.30 (95% CI: 0.06-0.70) per 100 persons-year among patients without preexisting cirrhosis. As the proportion of CHB patients with preexisting cirrhosis increased, the incidence density of HCC also increased gradually. Compared with other Nucleos(t)ide analogs (NAs) treatments, ETV and TDF treatments significantly lowered the risk of HCC, with hazard ratios (HRs) of 0.60 (95% CI: 0.40-0.90) and 0.56 (95% CI: 0.35-0.89), respectively. However, there was no difference in the incidence density of HCC between ETV and TDF treatments (HR = 0.92, 95% CI: 0.71-1.20) regardless of preexisting cirrhosis.

**Conclusion:**

ETV and TDF treatments were associated with significantly lower risks of HCC than other NAs treatments. However, no difference was observed between ETV and TDF treatments in the risk of HCC development regardless of preexisting cirrhosis among treatment-naïve CHB patients.

**Supplementary Information:**

The online version contains supplementary material available at 10.1186/s12885-022-09413-7.

## Background

Hepatitis B virus (HBV) infection remains a major global health problem, with 296 million people living with chronic hepatitis B (CHB) infection in 2019,with 1.5 million new infections each year [1]. CHB patients are at high risk of progression to cirrhosis and hepatocellular carcinoma (HCC) [2]. The Global Burden of Disease study estimated that HBV accounts for 33% of liver cancer-related deaths globally and 41% in Asia [3]. Nucleos(t)ide analogs (NAs) with a high barrier to HBV resistance, entecavir (ETV) or tenofovir disoproxil fumarate (TDF), are currently recommended as the first-line treatments for adults with immune-active CHB [4–6]. Long-term antiviral treatments are associated with a significantly lower HCC incidence in CHB patients by reducing HBV DNA concentrations [7]. However, HCC may still develop after antiviral treatment. Recent studies have suggested that there may be differences in the effects of ETV and TDF on the occurrence of HCC among CHB patients [8–11]. However, it is still controversial whether antiviral strategies affect HCC development in CHB patients [12].

Currently, meta-analyses on the effectiveness of TDF versus ETV on the incidence of HCC in CHB patients are derived from head-to-head comparisons among both antiviral treatment-naïve and antiviral therapy-experienced CHB patients [10, 13–15]. However, the efficacy of TDF and ETV treatment may be different in antiviral therapy-experienced CHB patients, since they may experience viral resistance before switch therapy [16]. Studies that directly compare the relative effect of ETV and TDF on the reduction of HCC development in antiviral treatment-naïve CHB patients are currently limited. Network meta-analysis can combine sources of both direct and indirect evidence [17] and provide estimates of the efficacy of multiple treatment regimens in antiviral treatment-naïve CHB patients [18].

In this study, we aimed to estimate the incidence of HCC in antiviral treatment-naïve CHB patients who were treated with ETV and TDF and compare the efficacy of two treatment regimens in HCC reduction through a systematic review and network meta-analysis.

## Methods

### Search strategy

We conducted a systematic literature search in the PubMed, Embase, China National Knowledge Infrastructure, and Wanfang databases until June 24, 2021. The search terms included the following: (‘chronic hepatitis B’ OR ‘hepatitis B virus infection, chronic’ OR ‘CHB’ OR ‘hepatitis B, chronic’ OR ‘Hepatitis B AND Chronic’) AND ((‘entecavir’ OR ‘Baraclude’ OR ‘ETV’) OR (‘Tenofovir disoproxil’ OR ‘Tenofovir’ OR ‘Viread’ OR ‘TDF’)) AND (‘Hepatocellular carcinoma’ OR ‘hepatocarcinoma’ OR ‘hepatic cellular cancer’ OR ‘HCC’). And the full search strategies for English databases were shown in the supplement. The reference lists from relevant articles were also screened. This study was registered in PROSPERO (No. CRD42019132954).

### Selection criteria

According PICOS framework, studies were included if they met the following criteria: 1) Patients: those on antiviral treatment-naïve chronic hepatitis B patients (HBsAg and/or HBV DNA positive and related symptoms and signs for at least 6 months, not treated with antiviral therapy previously); 2) Interventions and Comparisons: ETV monotherapy or TDF monotherapy; 3) Outcomes: HCC diagnosis met one of the following criteria: a) two typical imaging findings, such as those on ultrasound, enhanced computed tomography, and magnetic resonance imaging and lesion > 2 cm; b) one typical imaging finding, lesion > 2 cm, AFP > 400 ng/ml; c) liver biopsy was positive; and 4) Study design: randomized controlled trial or cohort study. Studies including CHB patients who had a history of aflatoxin exposure or coinfection with other viruses (HAV, HCV, HDV, HEV, HIV) or preexisting HCC were excluded.

### Data extraction and quality assessment

Two researchers independently assessed the eligibility of articles and extracted the required information using a standardized form. The differences were examined and settled through discussion with other authors. The information extracted from studies included the author names, publication year, study location, study design, sample size, characteristics of patients (HBeAg status, preexisting cirrhosis status), median duration of treatment, antiviral therapy and corresponding outcomes (number of patients with HCC, hazard ratio (HR)).

Quality was evaluated by Cochrane Collaboration’s tool for randomized controlled trials [19] and Newcastle-Ottawa Scale for cohort studies [20]. For randomized controlled trials, the following parameters were included when evaluating study quality: generation of the random sequence number, allocation concealment, blinding, data integrity, and selective reporting. For cohort studies, the following parameters were included when evaluating study quality: selection of the study population, comparability between groups, and measurement of the outcomes. The Newcastle-Ottawa Scale ranged from 0 to 9.

### Statistical analysis

R (version 3.6.1) was used for the statistical analysis. The pooled incidence and 95% confidence interval of HCC were calculated by the Freeman-Tukey double arcsine transformation method. The efficacy of the two treatment regimens in HCC reduction was compared through network meta-analysis. For the studies that included both entire cohorts and propensity score-matched cohorts, we prioritized the latter to reduce potential bias. For studies that reported only the HR, Review Manager 5.3 was employed to restore the number of outcomes. For studies with a value of 0, we adjusted the value to 0.01. Both the cumulative incidence and incidence density of HCC were calculated and compared. For the incidence density calculation, if the total persons-year follow-up was not reported, we estimated the total persons-year by multiplying the number of subjects by the mean or median treatment duration. Heterogeneity between studies was quantified with the *I*^2^ statistic (value greater than 50% was considered substantial heterogeneity). Subgroup analyses were conducted according to treatment duration and the preexisting cirrhosis status to explore the source of heterogeneity among studies. And the sensitivity analysis was conducted based on head-to-head comparison studies that reported the adjusted HRs by propensity score-matching analysis or multivariate Cox proportional hazard analysis.

## Result

### Study selection and characteristics

**Fig. 1:**
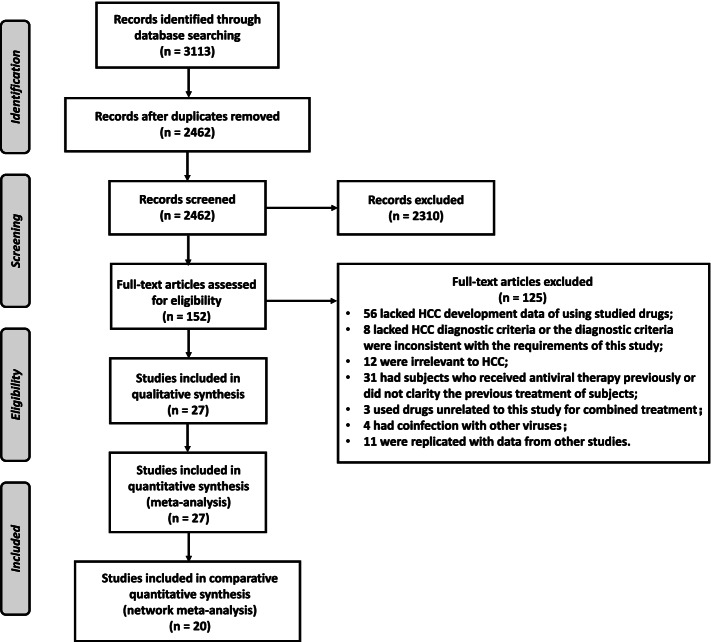
Flow chart of the literature retrieval process

A total of 3113 articles were initially identified. After excluding 651 duplicates, 2310 articles were excluded after reading the title and abstract. The full texts of the remaining 152 articles were reviewed, of which 27 were considered eligible for this systematic review. The study selection process is shown in Fig. 1.

The characteristics of the studies are shown in Table 1. All 27 studies were cohort studies and included a total of 52,373 chronic hepatitis B patients. Most studies (25, 93%) were from Asia, including South Korea, China, and Japan. The median treatment duration was 4.17 years (range: 0.92 to 7.29) for ETV, 3.18 years (range: 2.80 to 6.06) for TDF and 5.00 years (range: 0.92 to 6.80) for other NAs. Among all the subjects, males accounted for 62%. There were 16,490 (31%) HBeAg-positive patients, and 16,596 (32%) had preexisting cirrhosis. In total, 29, 13 and 7 studies separately analyzed HCC incidence among treatment-naïve CHB patients receiving ETV, TDF, and other NA (including lamivudine, telbivudine and adefovir) regimens. A total of 20 studies that provided comparisons of two or more treatments were used for the network meta-analysis.

**Table 1:** Characteristics of 27 studies included

Study (Year) (Ref.)	Country	No. of Male (%)	Age, Years^**a**^	No. of HBeAg positivity (%)	No. of cirrhosis (%)	Treatments	Median Treatment Duration	Person-years	HCC Cases	Sample size	Study Scored
Choi, J. 2019( [21]) Nationalwide cohort	South Korea	6802 (62)	49.1 ± 9.8	NA	2891 (26)	ETV	4.25	52,991	567	10,923	7
		6834 (63)	49.0 ± 9.8	NA	2919 (27)	TDF	3.00	53,030	350	10,923	
Coffin, C. S. 2014 ( [22])^b^	Canada	209 (65)	46 (38–55)	103 (32)	63 (20)	ETV	3.20	407	1	127	6
						TDF	3.20	423	3	132	
						Other NAs	3.20	203	7	63	
Güzelbulut, F. 2021 ( [23])	Turkey	186(72)	46.01 ± 14.06	53(21)	95(37)	ETV	4.68	1203	17	257	6
		234(60)	43.81 ± 13.40	102(26)	85(22)	TDF	3.54	1379	9	390	
Ha, I. 2020 ( [24])	South Korea	181 (61)	48 ± 16	161 (54)	39 (13)	ETV	3.00	894	11	298	8
		179 (60)	48 ± 14	174 (58)	39 (13)	TDF	3.00	894	22	298	
Hosaka, T. 2013 ( [25])	Japan	210 (66)	46 ± 12.1	135 (43)	79 (25)	ETV	3.30	1064	6	316	8
		210 (66)	46 ± 13.5	133 (42)	85 (27)	Control	7.60	2978	72	316	
		136 (75)	45 ± 10.7	71 (39)	85 (47)	Other NAs	6.80	1267	19	182	
Hsu, Y. C. 2018 ( [26])^b^	China	282 (73)	46 (36-55)	158 (41)	195 (51)	ETV	6.06	1357	20	224	6
						TDF	6.06	127	0	21	
						Other NAs	6.06	842	16	139	
Hsu, Y. C. 2020 ( [27])	China, Japan, South Korea, USA,	354(68)	44.12 ± 0.54	187(36)	107(21)	ETV	5.00	2600	19	520	8
		338(65)	44.88 ± 0.55	177(34)	105(20)	TDF	3.24	1685	11	520	
Kim, D. S. 2018 ( [28])^b^	South Korea	210 (63)	51.0 (42.8-57.0)	172 (51)	164 (49)	ETV	3.92	165	3	42	6
						TDF	3.06	894	22	292	
Kim, E. J. 2017 ( [29])	South Korea	366 (63)	51 ± 9	303 (52)	578 (100)	ETV	3.58	2069	81	578	5
Kim, G. A. 2017 ( [30])	South Korea	1288 (64)	47 ± 11	1168 (58)	815 (41)	ETV	4.82	9640	228	2000	5
Kim, J. H. 2017 ( [31])	South Korea	564 (64)	47.7 ± 10.7	483 (55)	443 (51)	ETV	4.50	3938	85	875	5
Kim, S. U. 2019 ( [32])	South Korea	793 (62)	48.6 ± 11.4	640 (50)	394 (31)	ETV	5.62	6187	93	1278	8
		794 (62)	48.2 ± 12.0	640 (50)	400 (31)	TDF	4.72	5457	91	1278	
Lee, J. 2016 ( [33])	South Korea	67 (66)	46.4 ± 11.2	71 (70)	36 (35)	ETV	3.17	323	7	102	5
Lee, S. W. 2019 ( [12])	South Korea	806 (59)	46.96 ± 11.75	814 (59)	465 (34)	ETV	5.00	6850	64	1370	8
		798 (58)	46.92 ± 11.13	807 (59)	464 (34)	TDF	3.03	4151	47	1370	
Li, L. 2012 ( [34])	China	29 (76)	54.7 ± 11.8	23 (61)	38 (100)	ETV	2.00	76	0	38	6
		24 (62)	52.2 ± 10.8	NA	39 (100)	Control	2.00	78	4	39	
		80 (69)	50.1	74 (64)	116 (100)	Other NAs	2.00	232	14	116	
Li, Y. 2013 ( [35])	China	14 (56)	36.32 ± 10.50	25 (100)	NA	ETV	5.00	125	3	25	6
		12 (52)	32.10 ± 0.21	23 (100)	NA	Other NAs	5.00	115	3	23	
Lim, Y. S. 2014 ( [36])	South Korea	1193 (67)	46.1 ± 10.1	1133 (63)	933 (52)	ETV	3.17	5687	137	1792	9
		1179 (66)	46.1 ± 10.9	1107 (62)	934 (52)	Other NAs	5.30	9503	234	1792	
Lin, T. C. 2018 ( [37])	China	NA	NA	NA	NA	ETV	5.24	938	13	179	5
Oh, H. 2020 ( [38])	South Korea	319(62)	49.2 ± 12.6	314(61)	238(46)	ETV	4.70	2425	29	516	8
		325(63)	49.0 ± 9.4	311(60)	224(43)	TDF	4.80	2477	37	516	
Ouyang, Y. 2011 ( [39])	China	22 (88)	50.1 ± 11.6	25 (100)	25 (100)	ETV	0.92	23	2	25	7
		78 (74)	46	105 (100)	105 (100)	Other NAs	0.92	97	9	105	
Sou, F. M. 2020 ( [40])	China	1018(73)	50 ± 17	491(35)	507(36)	ETV	7.29	10,190	133	1397	5
Shin, J. W. 2020 ( [41])	South Korea	365(62)	50 ± 11	365(62)	276(47)	ETV	4.86	2860	40	589	8
		358(61)	50 ± 11	354(60)	282(48)	TDF	3.58	2110	23	589	
Sohn, W. 2017 ( [42]) Testing cohort	South Korea	641 (65)	47.4 ± 10.5	556 (56)	389 (39)	ETV	2.10	2079	58	990	5
Sohn, W. 2017 ( [42]) Validation cohort	South Korea	669 (62)	46.6 ± 11.5	658 (61)	376 (35)	ETV	3.50	3749	85	1071	5
Su, T. H. 2016 ( [43])	China	345 (77)	50 (44-58)	150 (33)	450 (100)	ETV	4.00	1782	31	450	7
		345 (77)	51 (43-59)	131 (29)	450 (100)	Control	6.00	3021	115	450	
Wu, I. T. 2017 ( [44])	China	230 (73)	47 ± 12.3	172 (55)	94 (30)	ETV	4.08	1277	21	313	7
		74 (70)	47.1 ± 12.1	50 (47)	29 (27)	TDF	3.16	335	8	106	
Yip, T. C. 2020 ( [45])	China	2267 (49)	42.9 ± 12.7	2480 (53)	167 (4)	ETV	2.90	13,444	70	4636	8
		587 (49)	44.4 ± 13.1	625 (52)	37 (3)	TDF	2.80	3360	7	1200	
Yu, J. H. 2018 ( [46])	South Korea	272 (67)	53 (18–84)	212 (52)	148 (36)	ETV	5.83	2367	31	406	6
		104 (59)	49 (20–84)	104 (59)	77 (44)	TDF	2.80	493	7	176	

*NA* data not provided or unavailable, *No.* numbers, *HCC* hepatocellular carcinomam *Control* no treatment or expectant treatment, *ETV* Entecavir treatment, *TDF* Tenofovir disoproxil fumarate treatment; Other NAs, treatment with NAs except ETV and TDF (including Lamivudine, Telbivudine and Adefovir)

^a^Data are expressed as mean ± SD; otherwise, parenthesis indicates interquartile ranges

^b^Studies only provided baseline information of the entire population

### HCC incidence in treatment-naïve CHB patients receiving different treatments

**Fig. 2:**
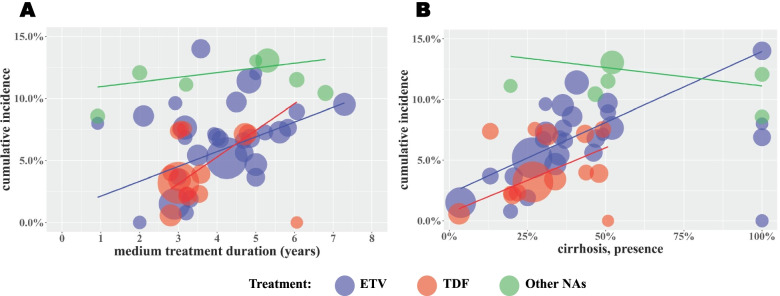
Bubble charts of the cumulative incidence by different (**A**) median treatment duration and (**B**) the proportion of CHB patients with preexisting cirrhosis subgroups. Trend lines fitted linearly represent the predicted HCC incidence according to different treatments. ETV, Entecavir; TDF, Tenofovir disoproxil fumarate; Other NAs, Nucleos(t)ide Analogues other than ETV and TDF (including Lamivudine, Telbivudine and Adefovir). The bubble size represents the sample size for each study

As shown in Fig. 2, the pooled estimates of the cumulative incidence of HCC among the ETV and TDF treatment groups increased with longer treatment durations and a higher proportion of CHB patients with preexisting cirrhosis. The median treatment duration and preexisting cirrhosis rate were the main sources of heterogeneity in the pooled estimation of HCC incidence (*R*^2^ = 68.11 and 64.29% in the ETV and TDF group estimations). To reduce bias in the estimation of HCC incidence, we calculated the pooled cumulative incidence and incidence density among patients with and without preexisting cirrhosis separately.

For patients with preexisting cirrhosis, the pooled estimates of cumulative incidence of HCC among the ETV treatment groups increased from 5.01% (95% CI: 1.32-10.49%) within 3 years of treatment to 14.21% (95% CI: 10.87-17.91%) after 5 years of treatment. For the patients treated with TDF, the cumulative incidence was 7.76% (95% CI: 5.46-10.42) at 3 to 4 years of treatment and 12.48% (95% CI: 5.86-21.11) at 4 to 5 years of treatment (Table 2).

**Table 2:** Pooled HCC incidence in CHB patients receiving different treatments via the meta-analysis

Treatment	Cumulative incidence (%, 95 ***CI***)				Incidence density (per 100 persons-year, 95% ***CI***)
	≤3 years	3-4 years	4-5 years	>5 years	
Total patients					
ETV	4.08 (0.88-9.03)	5.81 (3.52-8.61)	6.55 (4.84-8.49)	8.24 (7.34-9.18)	1.43 (1.14-1.75)
TDF	3.16 (1.12-6.14)	3.65 (2.21-5.42)	7.12 (5.97-8.37)	NE^a^	1.07 (0.74-1.46)
Other NAs	10.34 (6.60-14.78)	11.11 (4.35-20.23)	13.04 (1.81-30.45)	12.61 (11.22-14.07)	2.84 (1.86-4.00)
Patient with preexisting cirrhosis					
ETV	5.01 (1.32-10.49)	10.16 (6.91-13.93)	11.71 (10.72-12.73)	14.21 (10.87-17.91)	2.78 (2.21-3.40)
TDF	NA	7.76 (5.46-10.42)	12.48 (5.86-21.11)	NA	2.59 (1.51-3.96)
Other NAs	10.34 (6.60-14.78)	NA	21.28 (18.61-24.08)	24.49 (13.33-37.62)	4.81 (3.36-6.49)
Patient without preexisting cirrhosis					
ETV	0.66 (0.19-1.38)	1.79 (1.05-2.69)	2.78 (1.49-4.44)	3.04 (2.05-4.21)	0.49 (0.32-0.68)
TDF	0.09 (0.02-0.20)	NA	2.26 (1.17-3.68)	NA	0.30 (0.06-0.70)
Other NAs	NA	NA	NA	4.54 (3.31-5.94)	0.78 (0.56-1.02)

*CI* confidence interval, NA not available, *NE* not estimated

^a^Only one study contributed to these data, and none of the patient developed HCC during the follow-up

^*^If heterogeneity was greater than 50%, the results of the random effects model are reported in the table; otherwise, the results of the fixed effects model are reported

Similar trends were also observed in patients without preexisting cirrhosis; however, the cumulative incidence of HCC was significantly lower than that in patients with preexisting cirrhosis. The cumulative incidence of HCC increased from 0.66% (95% CI: 0.19-1.38) within 3 years of ETV treatment to 3.04% (95% CI: 2.05-4.21) after 5 years of ETV treatment. For patients receiving TDF treatment, the cumulative incidence was 0.09% (95% CI: 0.02-0.20) within 3 years of treatment and 2.26% (95% CI: 1.17-3.68) at 4 to 5 years of treatment (Table 2).

**Fig. 3:**
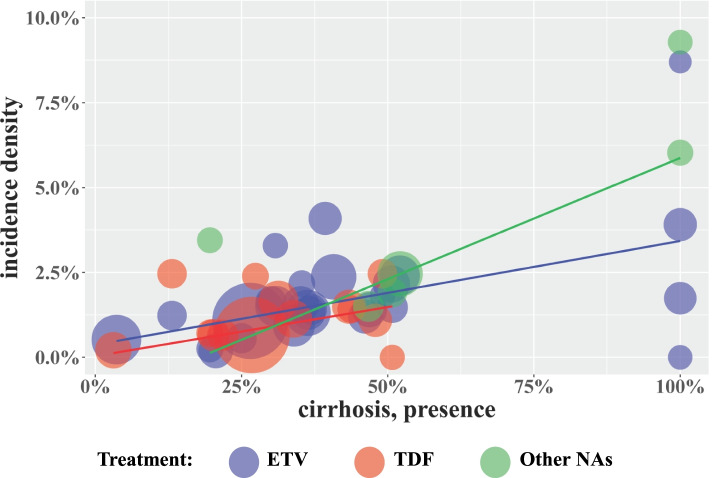
Bubble charts of incidence density according to preexisting cirrhosis proportion. Trend lines fitted linearly represent the predicted HCC incidence density according to different treatments. ETV, Entecavir; TDF, Tenofovir disoproxil fumarate; Other NAs, Nucleos(t)ide Analogues other than ETV and TDF (including Lamivudine, Telbivudine and Adefovir). The bubble size represents the sample size for each study

The incidence densities in the ETV and TDF treatment groups were 2.78 (95% CI: 2.21-3.40) and 2.59 (95% CI: 1.51-3.96) per 100 persons-year among patients with preexisting cirrhosis and 0.49 (95% CI: 0.32-0.68) and 0.30 (95% CI: 0.06-0.70) per 100 persons-year among patients without preexisting cirrhosis, respectively (Table 2). Treatment duration and the baseline cirrhosis rate were positively associated with HCC incidence in CHB patients treated with ETV or TDF (Fig. 2). As the proportion of CHB patients with preexisting cirrhosis increased, the incidence density of HCC also increased gradually (Fig. 3). In addition, the incidence of HCC among the ETV and TDF treatment groups was lower than that in the other NA treatment group (Table 2).

### Comparison of HCC risk in CHB patients receiving different treatments

**Fig. 4:**
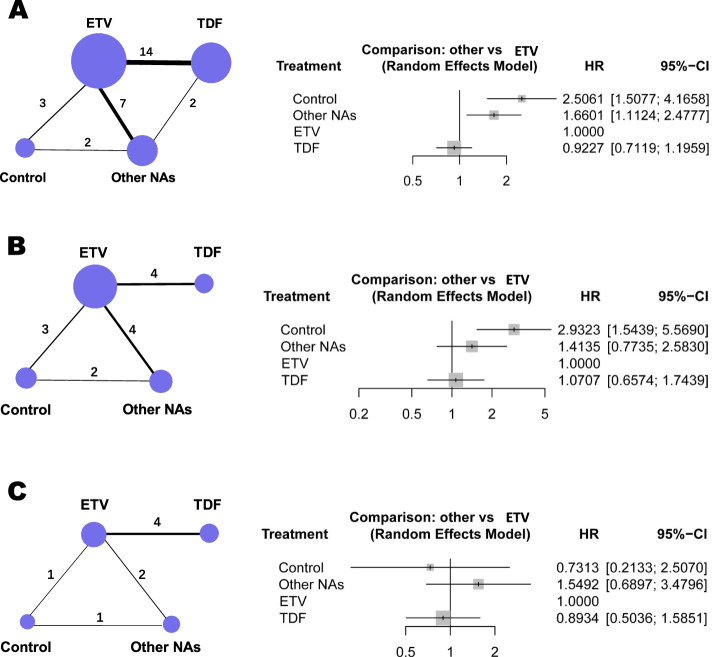
Network plot and forest plots from the network meta-analysis of HCC risk in CHB patients receiving different treatments. **A** Results of the total analysis. **B** Results of the subgroup analysis of patients with cirrhosis. **C** Results of the subgroup analysis of patients without cirrhosis. Control, no treatment or expectant treatment; ETV, Entecavir treatment; TDF, Tenofovir disoproxil fumarate treatment; Other NAs, Nucleos(t)ide Analogue treatments other than ETV and TDF (including Lamivudine, Telbivudine and Adefovir)

Twenty studies [12, 21–28, 32, 34–36, 38, 39, 41, 43–46] provided enough data for to compare HCC risk through network meta-analysis (Fig. 4). To reduce bias due to different treatment durations, the incidence density was used to compare the HCC risk in CHB patients receiving different treatments. Detailed results are shown in Fig. 4. Compared with other NA treatments, ETV and TDF treatments significantly lowered the HCC risk, with hazard ratios (HRs) of 0.60 (95% CI: 0.40-0.90) and 0.56 (95% CI: 0.35-0.89). However, there was no difference in the incidence density of HCC between ETV and TDF treatments (HR = 0.92, 95% CI: 0.71-1.20) regardless of preexisting cirrhosis. Similar results were observed in patients with and without preexisting cirrhosis (HR = 1.07, 95% CI: 0.66-1.74; HR = 0.89, 95% CI: 0.50-1.59).

### Sensitivity analysis

**Fig. 5:**
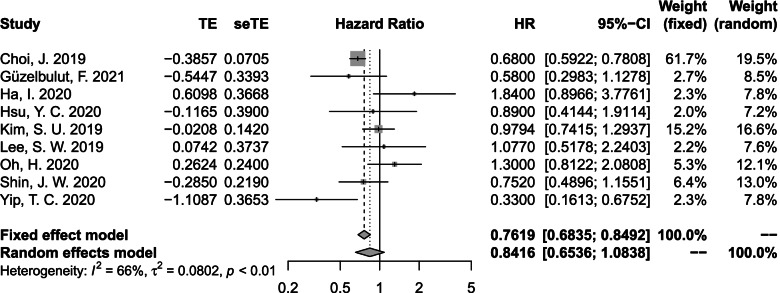
Pooled hazard ratio for HCC incidence between TDF and ETV treatments in CHB patients

We conducted sensitivity analysis based on six head-to-head comparison studies that reported the adjusted HRs by propensity score-matching analysis or multivariate Cox proportional hazard analysis (Fig. 5). Moderate heterogeneity was observed (*I*^*2*^ = 66%), so the result from the random effects model was appropriated. The pooled adjusted HR was 0.84 (95% *CI*: 0.65-1.08, *p* = 0.18).

## Discussion

Through this systematic review and network meta-analysis, we provided some important findings. First, ETV and TDF treatments were associated with a significantly lower risk of HCC than other NA treatments. Second, no difference was observed between ETV and TDF in the risk of HCC development among treatment-naïve CHB patients. Third, the proportion of CHB patients with preexisting cirrhosis was significantly associated with the incidence of HCC development, and this proportion should be balanced in future studies when comparison HCC risk according to ETV and TDF treatment.

A previous study demonstrated that other NA treatments can reduce HCC risk by 51% compared with no treatment [8, 9]. The current study showed that ETV and TDF treatments further reduced the incidence of HCC by more than 40%, from 2.84% per year in other NA-treated CHB patients to 1.10 ~ 1.71% per year in ETV- or TDF-treated patients. The annual incidence of HCC in CHB patients who received ETV or TDF was reported to range from 0.01% ~ 1.4 and 0.9% ~ 5.4 in noncirrhotic and cirrhotic patients, respectively [7]. In this study, the annual incidence densities in the ETV and TDF treatment groups were 0.49 and 0.30 per 100 persons-year among patients without preexisting cirrhosis and 2.78 and 2.59 per 100 persons-year among patients with preexisting cirrhosis, respectively. The downregulation of hepatic inflammation, reversal of fibrosis and reduction in regenerative stimuli at the tissue level, as well as reduction of HBx protein expression to levels insufficient to promote HCC development, may be the mechanisms by which NAs reduce HCC risk [47–49]. Achieving a virological response was significantly associated with the effectiveness of different NAs in HCC risk [7]. ETV and TDF were associated with a lower risk of viral resistance and higher virological response than other NAs, such as lamivudine and adefovir [2, 5, 50], which may contribute to the lower risk of HCC development after long-term treatment. In line with other studies, the residual risk of HCC in patients with preexisting cirrhosis was substantially higher than that in patients without preexisting cirrhosis [51]. Additionally, among patients with preexisting cirrhosis, the risk of HCC decreased over time with antiviral treatments. Studies have indicated that long-term antiviral treatments can result in the regression of liver fibrosis, which may lead to a reduction in HCC risk [52].

There was no significant difference in virological response between ETV and TDF treatments [44, 50, 53], and viral resistance to ETV and TDF is rare [54, 55]. Therefore, a similar incidence density of HCC was found among treatment-naïve CHB patients receiving ETV and TDF treatments in our study (1.43 vs 1.07 per 100 persons-year, HR = 0.92, 95% CI: 0.71-1.20). The results from recent meta-analyses on the comparison of the effectiveness of ETV and TDF for HCC reduction among both antiviral treatment-naïve and antiviral therapy-experienced CHB patients remain controversial [10, 13–15, 50, 56–61]. Our results were consistent with two meta-analyses [50, 56]. Meta-analyses [10, 57] that used raw data to compare the cumulative incidence of HCC among ETV and TDF treatment groups were inappropriate, since the duration of treatment was longer for ETV than for TDF in the majority of studies. The cumulative incidence of HCC among the ETV and TDF treatment groups increased with longer treatment durations. The remaining two meta-analyses used the log-transformed HR and 95% CI or incidence density to pool the overall HR for the comparison of ETV and TDF to reduce bias attributable to different treatment durations [13, 14]. These two meta-analyses included some studies on treatment-experienced patients (40% for Choi et al., 21% for Dave et al). Due to the earlier release of ETV than TDF, more patients experience viral resistance after switching to ETV than TDF, which may underestimate the effectiveness of ETV in HCC risk reduction [16]. In addition, HCC risk was not significantly different between ETV and TDF treatments in the unadjusted meta-analysis of 14 studies described by Dave et al. [14]. However, in his study, the risk of HCC among patients treated with ETV was higher than that among patients treated with TDF using a fixed-effects model with moderate heterogeneity (*P* = 0.04) from the available adjusted data of 7 studies. The sensitivity analysis indicated that the findings of Dave et al. were not robust. In our study, the findings are relatively robust, which are enhanced from following two aspects. First. compared to previous meta-analysis, this study is the first network meta-analysis comparing the risk of HCC between ETV and TDF monotherapy in antiretroviral treatment-naïve CHB patients that combines both direct and indirect sources of evidence. So this study included the largest target sample size. Second, this study assessed the bias introduced by inconsistent follow-up times and proportion of CHB patients with preexisting cirrhosis within patients treated with ETV and TDF monotherapy by using incidence density and subgroup analysis based on baseline cirrhosis status.

This study had some limitations. First, due to limitations of existing studies, only cohort studies were available, as randomized controlled trials are currently lacking. The selection bias between studies and differences in study design might have affected HCC risk in CHB patients treated with different antivirals. Second, due to the lack of sufficient studies, we were unable to distinguish additional subgroups, such as those with renal disease, different HBV DNA genotypes, obesity and smoking, to further compare the effects of different treatments on the risk of HCC in CHB patients with different subtypes. Third, medication adherence has a significant impact on the risk of HCC, and information on medication adherence was missing in most studies [62]. In addition, not all studies provided persons-year in the follow-up data, which may have led to bias in the estimation of the incidence density of HCC.

## Conclusion

In conclusion, antiviral treatment-naïve CHB patients treated with TDF or ETV had a lower HCC risk than those treated with other NAs. CHB patients with preexisting cirrhosis had a substantially higher residual risk of HCC. No significant difference was found in the risk of HCC development between antiviral treatment-naïve CHB patients treated with ETV or TDF. These results were derived from observational studies, so higher-quality randomized controlled trials may be needed in the future to enhance the reliability of the results.

## Supplementary Information

**Additional file 1.**

## Data Availability

Upon publication raw data from individual studies will be made available by the corresponding author to interested researchers requesting data for bona fide scientific purposes.

## Abbreviations

CHB: Chronic hepatitis B
ETV: Entecavir
HCC: Hepatocellular carcinoma
HRs: Hazard ratios
NAs: Nucleos(t)ide analogs
TDF: Tenofovir disoproxil fumarate
