# Supplementary material for: Risk of hepatocellular carcinoma in antiviral treatment-naïve chronic hepatitis B patients treated with entecavir or tenofovir disoproxil fumarate: a network meta-analysis
Source: BMC Cancer. 2022 Mar 17;22:287. doi: 10.1186/s12885-022-09413-7 (PMC8930063; doi:10.1186/s12885-022-09413-7)
Supplement: Supplementary file 1 — Additional file 1. [file 12885_2022_9413_MOESM1_ESM.docx]

**SEARCH STRATEGIES**

**Pubmed**

(((chronic hepatitis B) OR (hepatitis B, chronic) OR (Hepatitis B Virus Infection, Chronic) OR (CHB) OR ((Hepatitis B) AND Chronic)) AND ((Hepatocellular carcinoma) OR (hepatocarcinoma) OR (hepatic cellular cancer) OR (HCC)) AND ((Viread) OR (Tenofovir) OR (Tenofovir disoproxil) OR (TDF))) OR (((chronic hepatitis B) OR (hepatitis B, chronic) OR (Hepatitis B Virus Infection, Chronic) OR (CHB) OR ((Hepatitis B) AND Chronic)) AND ((Hepatocellular carcinoma) OR (hepatocarcinoma) OR (hepatic cellular cancer) OR (HCC)) AND ((ETV) OR (Baraclude) OR (entecavir)))

**Embase**

#10 #8 OR #9

#9 #2 AND #4 AND #5

#8 #3 AND #4 AND #5

#6 #1 AND #4 AND #5

#5 'chronic hepatitis b' OR 'hepatitis b, chronic' OR 'hepatitis b virus infection, chronic' OR 'chb' OR '(hepatitis b) and chronic'

#4 'hepatocellular carcinoma' OR 'hepatocarcinoma' OR 'hcc' OR 'hepatic cellular cancer'

#3 'viread' OR 'tenofovir' OR 'tenofovir disoproxil' OR 'tdf'

#2 'etv' OR 'baraclude' OR 'entecavir'

#1 'pegylated interferon alfa' OR 'pegasys' OR 'ifn' OR 'peg ifn'
